# Correlation between Odor Concentration and Volatile Organic Compounds (VOC) Composition of Environmental Tobacco Smoke (ETS)

**DOI:** 10.3390/ijerph13100994

**Published:** 2016-10-09

**Authors:** Miyuki Noguchi, Saya Tanaka, Kaede Watanabe, Akihiro Yamasaki

**Affiliations:** Faculty of Science and Technology, Seikei University, 3-3-1 Kichijoji-Kitamachi, Musashino, Tokyo 180-8633, Japan; noguchi@ejs.seikei.ac.jp (M.N.); t.saya.1019@gmail.com (S.T.); yamasakigroup@gmail.com (K.W.)

**Keywords:** tobacco smoke, air quality, odor, volatile organic compounds (VOC)

## Abstract

We examined the correlation between the odor concentration and the chemical composition of environmental tobacco smoke (ETS). Three types of ETS samples were prepared: secondhand smoke (SHS), thirdhand smoke (THS), and field ETS samples from an outside smoking area. The odor concentrations of the ETS, SHS, and THS samples were determined by the triangle-odor-bag method, and the chemical compositions were determined by proton transfer mass spectrometry. The odor concentration of the SHS samples was three or four orders of magnitude higher than that of the field ETS samples, and three orders of magnitude higher than that of the THS samples. The concentration ratios of the constituent chemicals in THS to those in SHS were about 10^−4^, corresponding to the ratio of the odor concentration. The concentration ratios of the constituent chemicals in the field ETS samples were much lower than the ratios of the odor concentrations. This suggests that the main contributing components to the odor of the field ETS samples are different from those in SHS and THS. The main contributors of the odor in the field ETS samples could be acetaldehyde, acetonitrile, acetic acid, and other unknown components with a mass-to-charge ratio (m/z) of 39 and 43.

## 1. Introduction

Environmental tobacco smoke (ETS) is a mixture of mainstream tobacco smoke exhaled by smokers and sidestream smoke generated by burning tobacco. ETS contains several thousand chemicals in different forms, such as vapors, particulates, and adsorbed particulates [1,2,3,4]. Some of these chemicals are known to cause adverse human health effects, such as asthma and lung cancer. Because of public concerns about these health effects, smoking in public spaces has been banned in many countries, and strict separation of smoking areas is deployed to prevent the unnecessary exposure of non-smokers to secondhand smoke (SHS). Apart from the direct health effects caused by SHS, some of its components possessing a relatively high boiling point adsorb to airborne particles, walls, and cloths; the adsorbed compounds continue to be emitted into the air for a long time. The environmental smoke thus generated is termed thirdhand smoke (THS) [5,6,7,8,9], and concerns have been raised about exposure to THS [7,8,9], the constituent components of which are different from those of SHS owing to chemical reactions. A number of studies have been published on the chemical composition of ETS, including SHS and THS [10,11,12,13]. Characterization of the chemicals involved would assist in evaluating the fundamental risks of human exposure to SHS or THS caused by these hazardous compounds [12]. Some compounds contained in ETS, however, have extremely low olfactory threshold values; for example, the odor threshold value of pyridine is about 3.7 ppb [13]. Such malodorous compounds in ETS may contribute to psychological effects in passive smokers; passive smokers that notice the malodor of ETS should feel offended, even if the concentration levels are below the threshold values that affect human health. Since smoking is not strictly prohibited in public spaces in most countries, smokers and/or providers of smoking places for smoking should be conscious about the detection of tobacco odor by non-smokers. Thus, reducing odor from ETS is a key step in maintaining comfortable living conditions through the separation of smoking areas [13]. Despite intensive work analysis of the constituent components of ETS [4,11,14,15,16,17,18], there are very few studies focusing on the relationship between odor and the concentrations of odorous chemicals in ETS. Identifying the malodorous components in ETS could lead to effective countermeasures to reduce ETS malodor. Because volatile organic compounds (VOCs) contained in ETS are the main sources of odor [19], the contributions of each VOC component to ETS odor should be evaluated quantitatively. However, because of the extremely large numbers of chemicals contained in ETS, it might be difficult to identify the major or main contributor chemicals of the malodor of ETS. One possible and practical approach is to correlate the composition of malodorous components with the odor concentration in ETS. If a given component dominantly contributes to ETS odor, a correlation should be found between the odor concentration and the concentration of that particular chemical. In this study, we conducted simultaneous measurements of odor and VOC composition for various ETS samples, and the correlations between odor concentration and chemical composition are demonstrated based on the experimentally observed results.

## 2. Experimental

### 2.1. Preparation of ETS Samples

Three types of ETS sample gases were prepared for the measurements: (1) secondhand smoke (SHS); (2) thirdhand smoke (THS) desorbed from a plastic bag; (3) air sample from a smoking area (field ETS). The preparation or sampling methods of these ETS samples are as follows.

**Figure 1:**
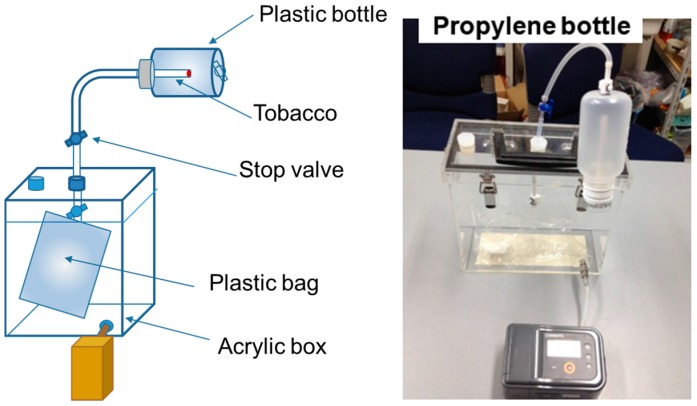
Schematic drawing and a photograph of the tobacco smoke generator.

**Figure 2:**
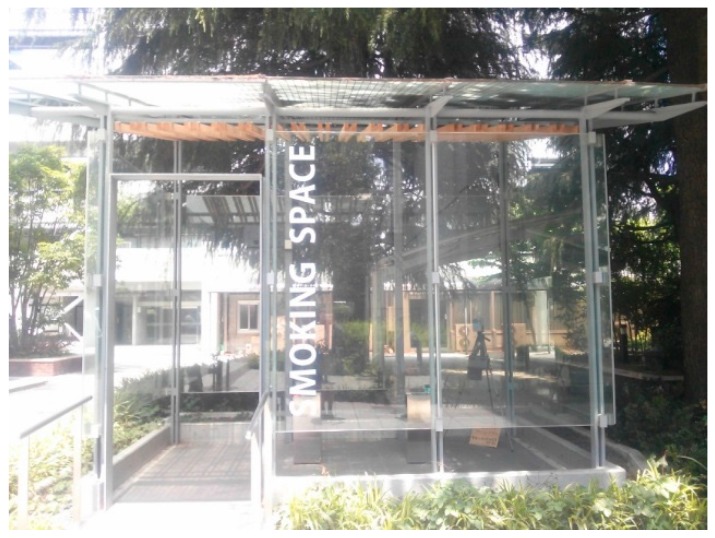
A photograph of the smoking space at Seikei University.

(1) The SHS sample was prepared by using an apparatus schematically shown in Figure 1. A tobacco cigarette (Mild Seven, produced by Japan Tobacco Inc., Tokyo, Japan), was burned in a polypropylene bottle, connected by a plastic tube to a plastic bag (volume 2.0 L) placed in an acrylic box (inner volume 6.0 L). The box was degassed beforehand with a vacuum pump with an evacuation rate of 1.0 L/min. The tobacco smoke generated in the polypropylene bottle was introduced into the plastic bag by the pressure difference between the bottle and the box. After filling the bag with smoke, the valve connecting the bottle with the bag was closed, and the SHS was sampled. In this study, only side stream smoke (SSS) was collected as the SHS sample gas. The composition of the chemicals in SSS may depend on the smoking conditions, and the chemical composition of SSS collected in this method may be different from that in the field ETS.

(2) The THS was sampled as follows. After sampling SHS by the method mentioned in (1), we evacuated the bag by vacuum pump. Clean dry air was then introduced into the plastic bag from a gas cylinder. The plastic bag was left under ambient conditions for 30 min to allow for desorption of volatile components from the bag itself into the clean air. A blank test was performed without introduction of SHS, and the gaseous components emitted from the bag surface were recorded in advance. Note the smoke components may react with the surface of the plastic bag, and the THS sample collected was specific to the plastic bag used.

(3) The field ETS sample was collected at a smoking space located on the campus of Seikei University, Musashino, Tokyo, Japan. The smoking space is a semi-open place surrounded by glass-plate walls and a glass-plate roof. Natural ventilation may take place through the open space between the glass plates without ventilation equipment (Figure 2). The volume of the smoking area was approximately 25 m^3^ (2.4 m × 3.9 m × 2.7 m). The field ETS sample in the smoking space was collected in an odor sampling bag (volume 3.0 L) by collecting the ambient air of a given place for 6 s with a flex pump with a flow rate of 30 L/min. As a control, ambient air was sampled by the same method at a location far from the smoking area, where the odor of tobacco smoke was not sensed. The field ETS sampling was conducted three times in July 2013 over different dates and under different conditions, with a varying number of smokers (6, 1, and none) in the smoking area.

### 2.2. Odor Concentration Measurements

The odor concentrations of the ETS samples were measured with a sensory test method termed the triangle-odor-bag method [20]. This method has been widely used as a standard odor measurement method in Japan, and the results of our odor measurements were consistent with the results from dynamic olfactometry [21].

The measurement procedure was as follows. Six or more volunteers were employed as a panel group. In this study, six panelists, three female and three male college students in their early twenties, were employed. They had no special training on the odor detection, and medical ethical permission was obtained prior to the odor measurement. Three identical bags were prepared for each panelist. Two of the three bags contained clean air, and the third contained a sample gas to measure odor concentration. The contents of the three bags were sniffed by a panelist to sense the odor without any information about the content. The panelist was asked to point out the bag containing the sample gas. If the panelist selected the correct bag, the procedure moved to the next stage: Three identical bags were presented, two of which contained clean air and the third contained a sample gas diluted with clean air to a pre-determined dilution ratio. The sniff test was then repeated. If the panelist indicated the correct bag, the test moved to the next stage, using the sample gas with a higher dilution ratio. The test was repeated if the panelist failed to sense the sample gas or selected one of the controls. The parameter, *X_i_*, was defined by the following formula,

(1) $$X_{i}=\frac{\log M_{1}+\log M_{0}}{2}$$

where *M*_1_ was the maximum (final) dilution ratio at which the panelist correctly indicated the bag containing the sample gas, and *M*_0_ was the minimum dilution ratio at which the panel group failed to indicate the bag containing the sample gas. The mean value of *X_i_* was calculated based on the four panelists who gave moderate values out of six panelists,

(2) $$\bar{X}=\frac{\sum_{i}X_{i}}{4}$$

Then the odor concentration, *Z*, of the sample gas, was finally given by,

(3) $$Z=10^{\bar{X}}$$

The odor index, OI, was defined by using the odor concentration,

(4) $$\mathrm{OI}=10\log10^{\bar{X}}=10\bar{X}$$

### 2.3. VOC Concentration Measurement

The concentrations of VOCs composing the gas sample were analyzed by proton transfer mass spectrometry (PTR-MS) (Ionicon Analytik, Innsbruck, Austria). PTR-MS (Proton Transfer Reaction—Mass Spectrometry) is a simultaneous real-time monitoring of volatile (organic) compounds (VOCs) without sample preparation in very low concentrations. The sample gas was introduced to the apparatus and reacted with an excess amount of hydronium ions (H_3_O^+^). Protons from the hydronium ions were transferred to the target chemicals (proton transfer reaction, PTR) to form ionic compounds. These ionic compounds were then introduced to mass spectrometry to determine the concentrations. Most of gaseous organic chemicals could be detected by the PTR-MS with a detection limit of about 1 ppt (parts per trillion). The mass spectrometry covers the m/z range from 22 to 220. The operating conditions were as follows. For the calculation of the VOC concentration, we followed the standard or default method provided by the manufacturer (IONICON Analytik [22]) and assumed that the rate constant of the proton transfer reaction was set constant at *k* = 2.0 × 10^−9^ cm^3^·s^−1^. The pressure, temperature, and voltage of the drift tube were 2.15 mbar, 70 °C, and 600 V, respectively. A quadrupole mass spectrometer was used. The particulates in the sample gas did not affect the concentration analysis. The effect of moisture on the analysis was negligible for all the analyses. We ignored the fragmentation of mass spectra based on the manufacturer’s data Ion fragmentation was not considered according to the manufacture’s data [22].

## 3. Results and Discussion

### 3.1. Odor Concentration and Odor Index

Table 1 shows the results of the measurements for the odor concentrations and the odor indices of the various ETS sample gases.

The SHS samples displayed the highest odor concentration, 2 × 10^9^, and the corresponding odor index was 94. The THS samples had an odor concentration that was four orders of magnitude lower at approximately 10^5^, equivalent to an odor index of 55. The field ETS samples showed an odor concentration in the range of 10^5^ to 10^6^, and the highest odor concentration was observed for the field ETS sample collected in the presence of six smokers, the highest number of smokers present during sampling. The odor indices of the field ETS samples were in the range of 55–65. The regulated odor index in a living environment in Japan is in the range of 10–21, which is much lower than the ETS samples tested in this study.

### 3.2. Composition of the Sample Gases Analyzed by PTR-MS

Table 2 shows the concentrations of the components in the ETS samples detected by PTR-MS in order of concentration. The chemicals identified are shown in Table 2 with a superscript. Note that we cannot exclude the possibility that the concentrations are the sum of isobaric and isomeric compounds. In the SHS model, isoprene showed the highest concentration, followed by nicotine. The concentrations of these chemicals are much higher than the published threshold values of the odor detection determined by the triangle-odor-bag method, which is 1.5 ppb for acetaldehyde, 0.5 ppm for formaldehyde, 42 ppm for acetone, 48 ppb for isoprene, and 11 ppb for nicotine [20].

**Figure 3:**
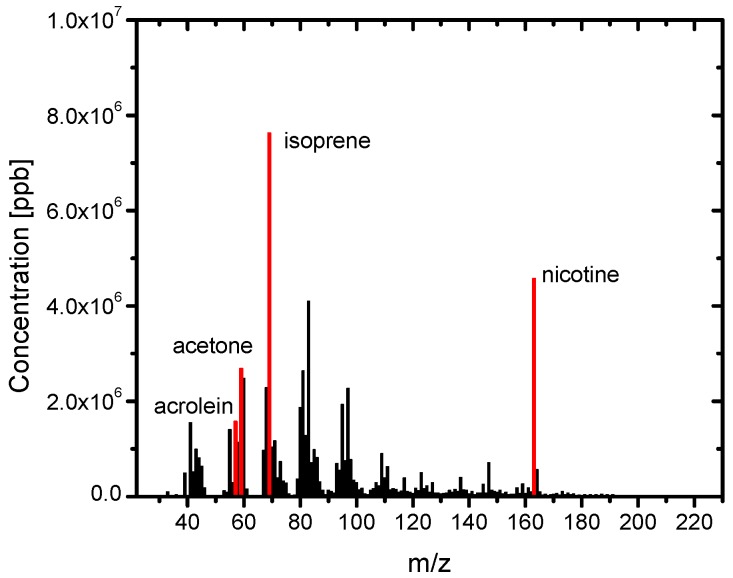
Proton Transfer Reaction-Mass (PTR-MS) spectra of the SHS samples (no dilution).

**Figure 4:**
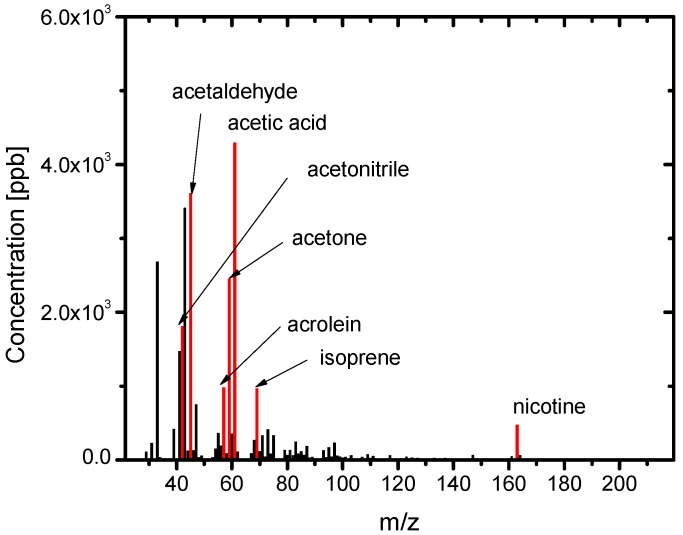
PTR-MS spectra of the THS sample.

**Figure 5:**
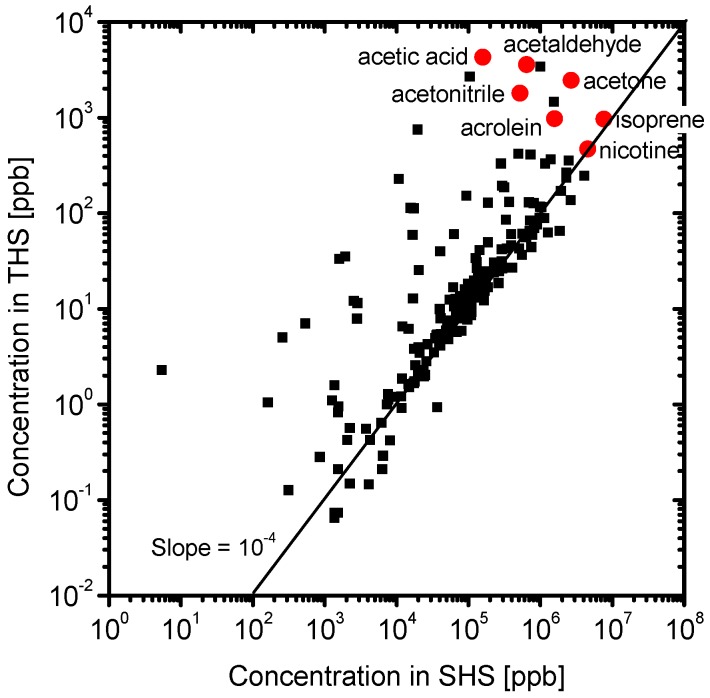
Correlation between the chemical concentrations in SHS and in THS. Red points indicate identified chemicals with the highest concentrations.

**Figure 6:**
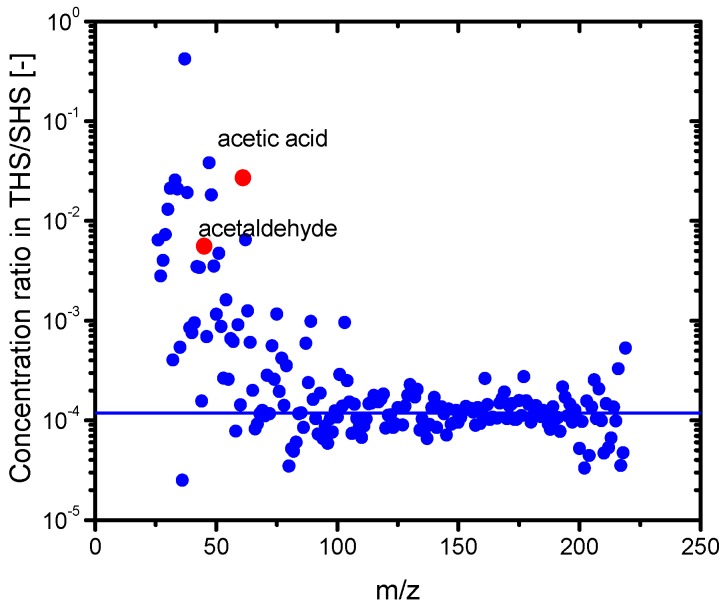
Concentration ratio of each chemical in THS/SHS plotted against the mass-to-charge ratio (m/z). The solid line denotes the ratio of the odor concentration of THS to SHS.

Figure 3 shows the PTR-MS spectra of the SHS samples. Various chemicals were detected. The identified chemicals with the highest concentrations are acrolein (m/z = 57), acetone (m/z = 59), isoprene (m/z = 69), and nicotine (m/z = 163). Figure 4 shows the PTR-MS spectra of the THS samples desorbed from the wall of the plastic bag into the clean air. Although the sample SHS was degassed from the plastic bag, a number of chemicals were detected in the THS. The concentration level of THS chemicals was 10^3^ ppb, which is approximately three to four orders of magnitude lower than those in the SHS samples. The constituent components with higher concentrations include acetonitrile, acetic acid, acetaldehyde, acetone, acrolein, and isoprene, which are oxygen-containing compounds with relatively low molecular weights. To clarify the difference between the SHS and THS samples in terms of the chemical concentrations, the concentration of each chemical in THS was plotted against its concentration in SHS (Figure 5). We observed a linear correlation between the concentrations of chemicals in the SHS and THS samples. Most chemicals are on a line with a slope of 10^−4^, including nicotine and isoprene, which corresponds to the ratio of the odor concentration of SHS samples to that of THS samples. Some components displayed a departure from the correlation line, namely acetic acid and acetaldehyde; the ratios of these components in THS were much higher than their ratios of odor concentration. To clarify the deviation of these chemicals from the correlation line, we plotted the ratio of the concentrations in THS and in SHS against the m/z (Figure 6). In general, chemicals with m/z values higher than 80 had a ratio of approximately 10^−4^, while chemicals with m/z values lower than 80 had a ratio higher than 10^−4^. This is presumably because of the fact that low molecular weight chemicals have, in general, lower boiling points, and consequently a higher tendency to desorb. The contributions of the lower m/z chemicals to the odor concentration are, however, not significant; instead, the odor concentration of the THS samples should be determined mainly by the chemicals possessing m/z values higher than 100.

**Figure 7:**
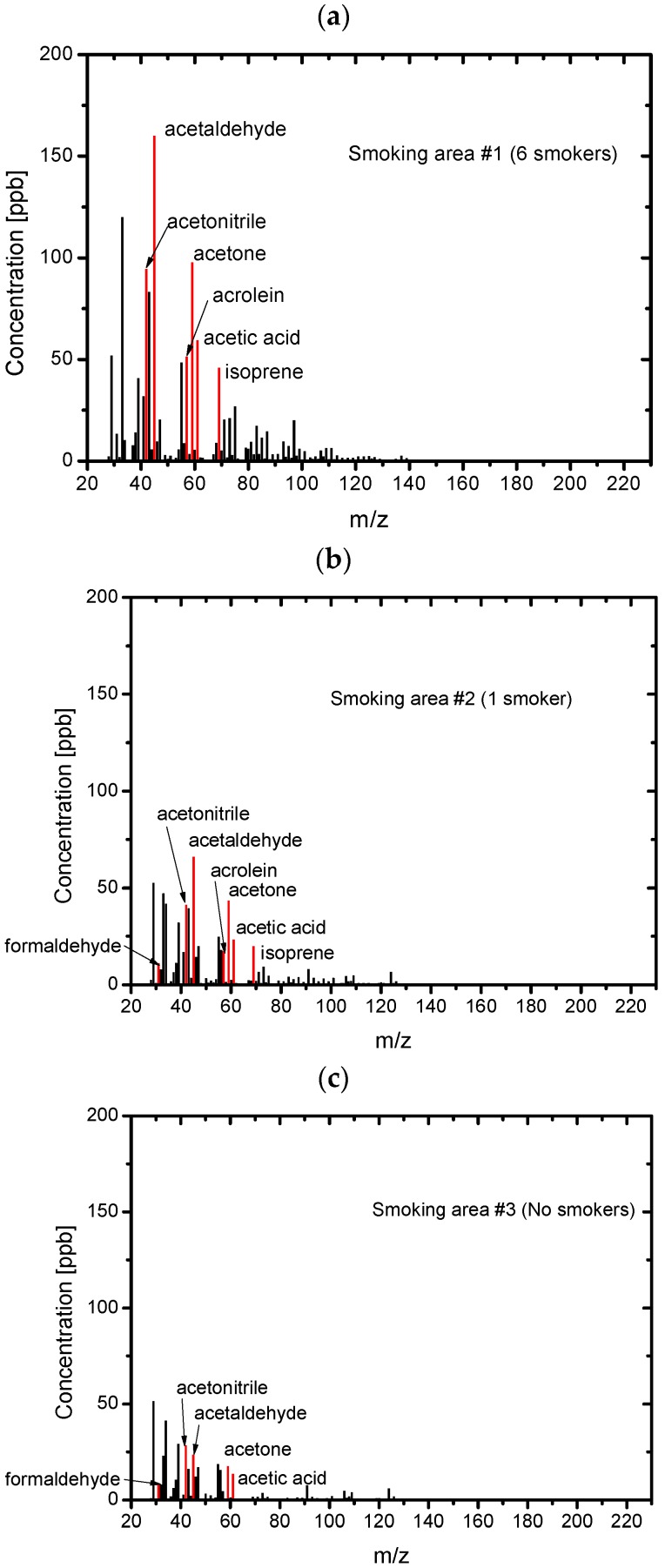
PTR-MS spectra of the field ETS samples. (**a**) Smoking area #1 with six smokers; (**b**) Smoking area #2 with one smoker; (**c**) Smoking area #3 with no smokers.

Figure 7 shows the PTR-MS spectra of the field ETS samples collected in the smoking area. The absolute values of the concentrations, approximately 100 ppb for the higher-concentration chemicals, were much lower than the SHS or THS samples. In general, the higher concentrations were observed for samples collected in the presence of six smokers. It should be noted that the order of the odor concentrations of the field ETS samples was similar to the order of the odor concentrations in THS.

**Figure 8:**
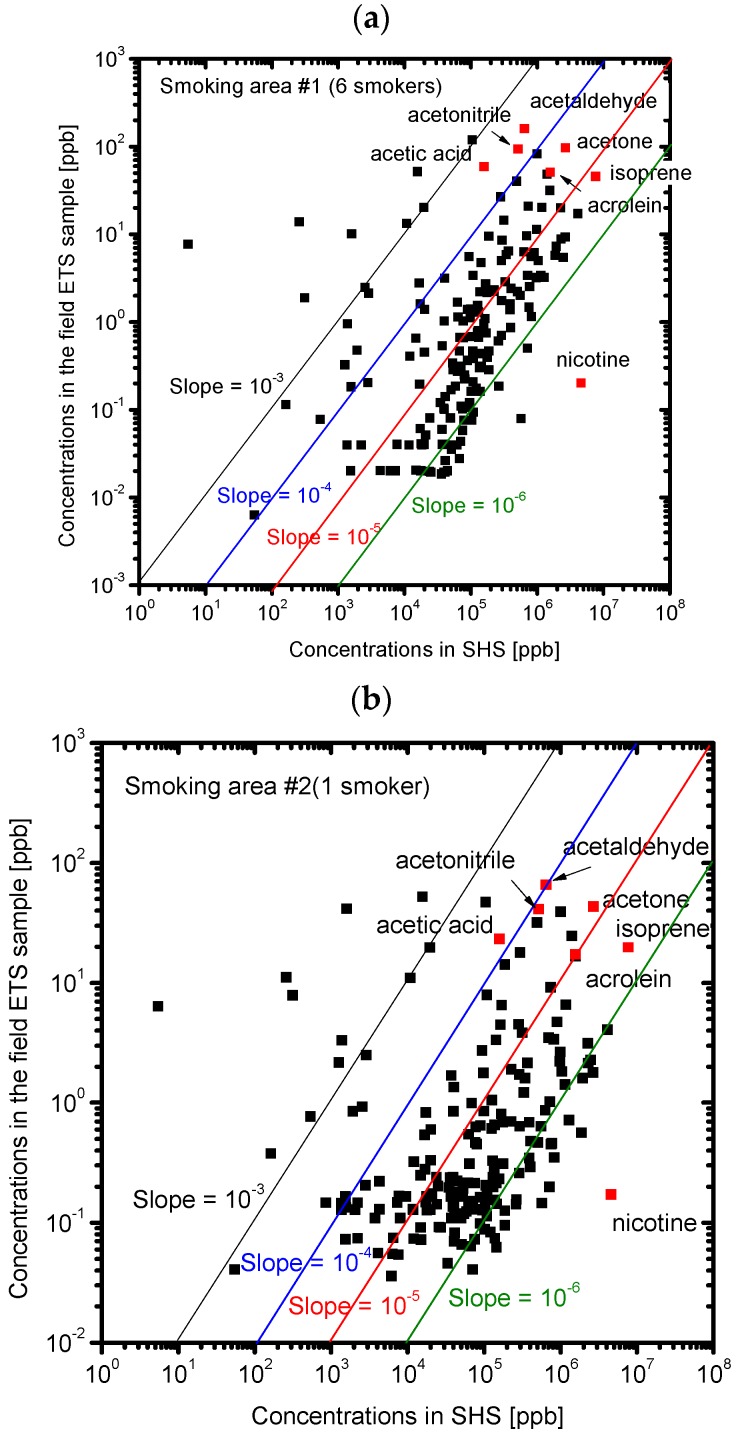

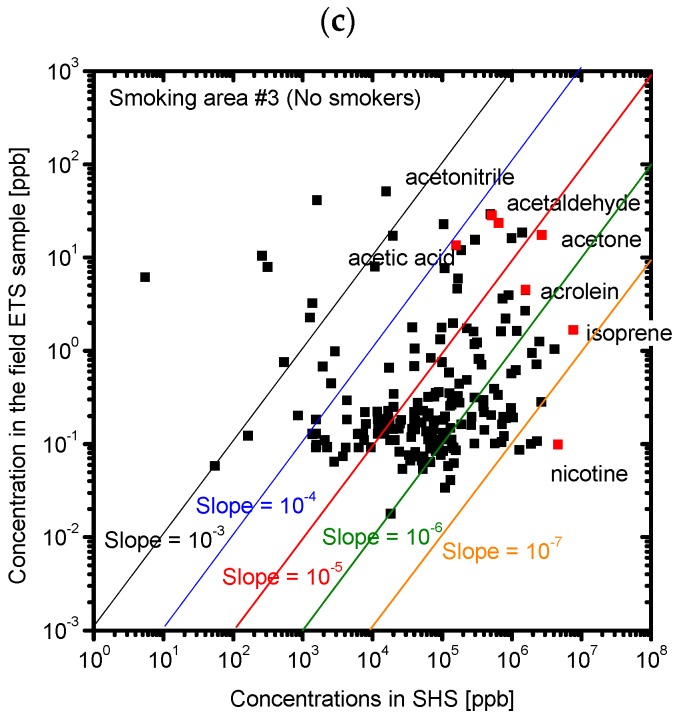
Correlation between the concentrations of chemicals in SHS and those in the field ETS samples. Red points indicate the identified chemicals with the highest concentrations. (**a**) Smoking area #1 with six smokers; (**b**) Smoking area #2 with one smoker; (**c**) Smoking area #3 with no smokers.

**Figure 9:**
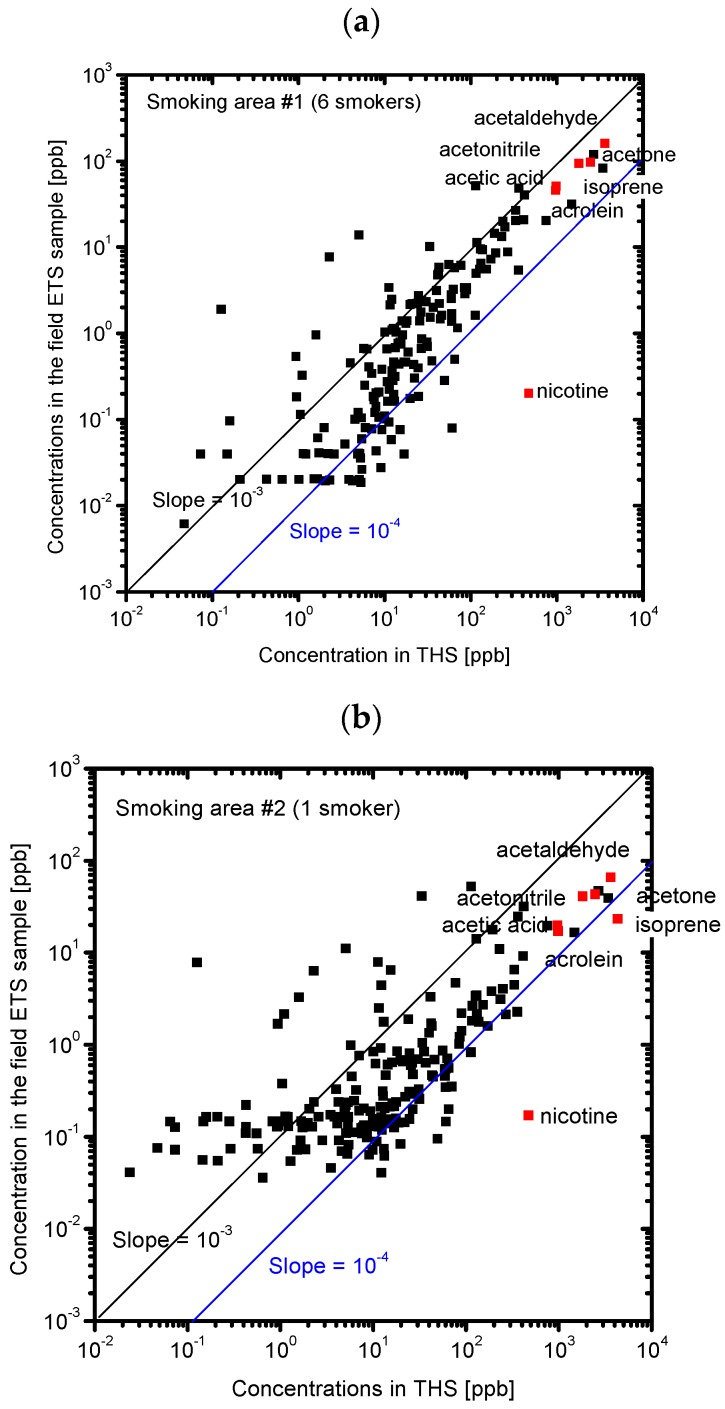

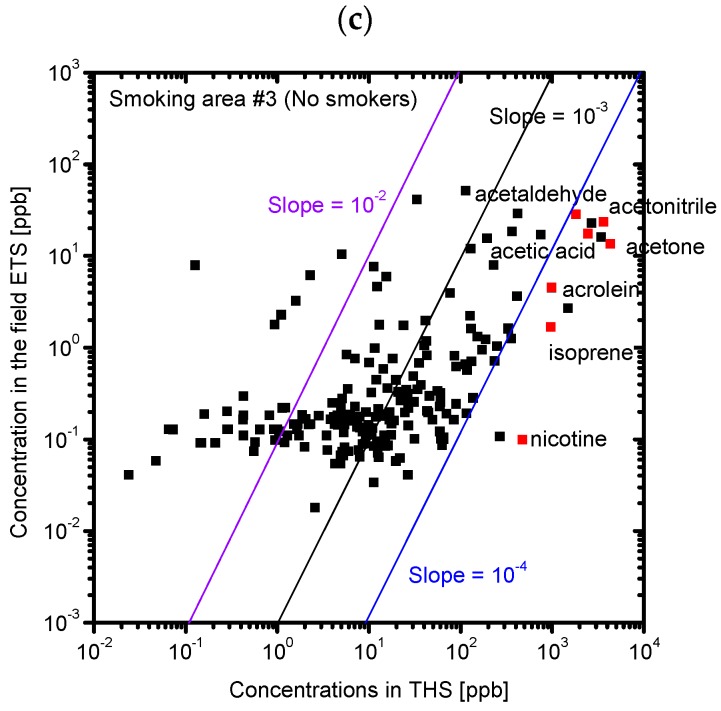
Correlation between the concentrations of the chemicals in THS and those in the field ETS samples. Red points indicate the identified chemicals with the highest concentrations. (**a**) Smoking area #1 with six smokers; (**b**) Smoking area #2 with one smoker; (**c**) Smoking area #3 with no smokers.

Figure 8 shows the correlation between the concentrations of the chemicals in the field ETS samples and the SHS samples. A correlation was observed for each field ETS sample, but a better correlation was observed for the samples taken when six smokers were present. Although the ratios of the odor concentration of the field ETS samples to that of the SHS samples are about 10^−3^ to 10^−4^, the observed ratios of the concentrations, which can be represented by the slope of the correlation line in Figure 8, were much lower, at about 10^−5^ to 10^−6^. This result suggests that the odor of the field ETS samples are determined by the different chemicals that determine the odor in SHS and THS. We assume that the odor concentration of the field ETS samples is determined by the constituent chemicals whose concentration ratio to that in the SHS samples is equivalent to the ratio of the odor concentration. The chemicals whose concentration ratios are close to the odor concentration ratio, such as acetaldehyde, acetonitrile, acetic acid, and other unknown components (m/z = 39, 43) would significantly contribute to the odor of the field ETS samples. Figure 9 shows the correlation between the concentrations of the chemicals in the field ETS samples and those in the THS samples. Although it is difficult to compare the strength of the correlations, better correlations were observed for the concentrations of the chemicals in the field ETS plotted against those in THS rather than those in SHS, especially for the ETS case without smokers. This finding suggests that the compositions of the field ETS samples are more similar to the composition of the THS than to that of the SHS. Because the field ETS samples represent a mixture of the SHS and the THS in the smoking area, the higher contribution of the SHS can be attributed to the field ETS with the increasing number of smokers.

## 4. Conclusions

In summary, we found a linear correlation between the concentrations of the constituent chemicals, represented by m/z, of the SHS and THS samples. The ratios of the concentrations were equal to the ratio of the odor concentrations determined by the triangle-odor-bag method, especially for the high molecular components (m/z > 80). Correlations were also observed between the concentrations of the constituent chemicals of the field ETS samples and the SHS and THS samples. The concentration ratios were, however, orders of magnitude smaller than the ratio of the odor concentrations. This suggests that the chemicals mainly attributable to the odor concentrations are different for the field ETS samples.

In this study, all constituent chemicals in the samples were represented by m/z values detected by PTR-MS. Only a few components were identified; most of the constituent chemicals were not identified. Future work will focus on identifying and ranking the contributions of these chemicals for effective measures of malodor caused by ETS.

## Figures and Tables

**Table 1:** Odor concentrations of Environmental Tobacco Smoke (ETS) sample gases by the triangle-odor-bag method.

Sample	Odor Concentration	Odor Index
Second Hand Smoke (SHS)	2.7 × 10^9^	94
Third Hand Smoke (THS)	3.2 × 10^5^	55
Field ETS #1 (18 July); 6 smokers	3.2 × 10^6^	65
Field ETS #2 (19 July); 1 smoker	3.2 × 10^5^	55
Field ETS #3 (19 July); no smokers	3.2 × 10^5^	55

**Table 2:** Chemicals and their concentrations in the sample gases with the twenty highest concentrations.

m/z	Second Hand Smoke (SHS)	Third Hand Smoke (THS)	Smoking Area #1	Smoking Area #2	Smoking Area #3
			6 Smokers	1 Smoker	No Smokers
	Concentration (ppb)	Concentration (ppb)	Concentration (ppb)	Concentration (ppb)	Concentration (ppb)
29	-	-	52.0	52.4	51.5
31 ^a^	-	-	-	11	8.0
32	-	-	-	7.9	7.9
33	-	2682.8	120.2	47.1	23.0
34	-	-	-	41.7	41.3
37	-	-	-	-	6.2
38	-	-	13.9	11.1	10.5
39	-	418.5	40.6	32	29.2
41	1.55 × 10^6^	1474.1	31.8	16.7	-
42 ^b^	-	1806.6	94.4	41.2	28.5
43	9.95 × 10^5^	3412.6	83.1	39.4	16.1
45 ^c^	-	3606.7	160.1	66	23.5
46	-	-	-	-	12.0
47	-	750.3	20.4	19.7	17.1
55	1.40 × 10^6^	363.3	48.5	24.7	18.6
56	-	-	-	17.9	-
57 ^d^	1.57 × 10^6^	976.9	51.3	17.3	15.6
58	1.14 × 10^6^	-	-	-	-
59 ^e^	2.69 × 10^6^	2451.9	97.6	43.4	17.6
60	2.48 × 10^6^	355.9	-	-	-
61 ^f^	-	4296.1	59.3	23.3	13.5
67	9.70 × 10^5^	-	-	-	-
68	2.29 × 10^6^	267.7	-	-	-
69 ^g^	7.63 × 10^6^	964.1	45.7	19.9	-
70	1.04 × 10^6^	-	-	-	-
71	1.17 × 10^6^	332.4	20.4	-	-
73	-	411.1	21.0	9.2	-
75	-	330.5	26.7	-	-
80	1.87 × 10^6^	-	-	-	-
81	2.64 × 10^6^	-	-	-	-
82	1.28 × 10^6^	-	-	-	-
83	4.10 × 10^6^	248.2	17.2	-	-
85	9.81 × 10^5^	-	-	-	-
87	-	-	14.5		
91	-	-	-	7.9	7.7
95	1.93 × 10^6^	-	-	-	-
97	2.27 × 10^6^	235.6	20.0	-	-
106	-	-	-	-	4.7
124	-	-	-	-	6.0
163 ^h^	4.58 × 10^6^	468.8	-	-	-

m/z, mass-to-charge ratio. Chemicals with asterisks are identified probably as, ^a^ formaldehyde; ^b^ acetonitrile; ^c^ acetaldehyde; ^d^ acrolein; ^e^ acetone; ^f^ acetic acid; ^g^ isoprene; ^h^ nicotine.
